# Six-month follow-up of functional status in discharged patients with coronavirus disease 2019

**DOI:** 10.1186/s12879-021-06970-3

**Published:** 2021-12-20

**Authors:** Hou-wei Du, Shuang-fang Fang, Sang-ru Wu, Xiao-ling Chen, Jun-nian Chen, Yi-xian Zhang, Hua-yao Huang, Han-han Lei, Rong-hua Chen, Xiao-bin Pan, Xiao-qing Li, Pin-cang Xia, Zhen-yang Zheng, Ling-Luo, Hai-long Lin, Li-min Chen, Nan Liu, Fujian Medical Team Support Wuhan for COVID19

**Affiliations:** 1 Department of Neurology, Fujian Medical University Union Hospital, 29 Xinquan Road, Gulou District, Fuzhou, 35000 China; 2 Institute of Clinical Neurology, Fujian Medical University, Fuzhou, China; 3 Department of Infectious Disease, Fujian Medical University Union Hospital, Fuzhou, China; 4 Department of Critical Care Medicine, Fujian Medical University Union Hospital, Fuzhou, China; 5 Department of Rehabilitation, Fujian Medical University Union Hospital, Fuzhou, China; 6 Department of Critical Care Medicine, Fujian Provincial Hospital South Branch, Fuzhou, China; 7 Fujian Center for Disease Control and Prevention, Fuzhou, China; 8 Department of Radiology, Fujian Medical University Union Hospital, Fuzhou, China; 9 Department of Respiratory Medicine, Fujian Medical University Union Hospital, Fuzhou, China

**Keywords:** Coronavirus disease 2019, Functional outcome, Follow-up

## Abstract

**Background:**

The long-term functional outcome of discharged patients with coronavirus disease 2019 (COVID-19) remains unresolved. We aimed to describe a 6-month follow-up of functional status of COVID-19 survivors.

**Methods:**

We reviewed the data of COVID-19 patients who had been consecutively admitted to the Tumor Center of Union Hospital (Wuhan, China) between 15 February and 14 March 2020. We quantified a 6-month functional outcome reflecting symptoms and disability in COVID-19 survivors using a post-COVID-19 functional status scale ranging from 0 to 4 (PCFS). We examined the risk factors for the incomplete functional status defined as a PCFS > 0 at a 6-month follow-up after discharge.

**Results:**

We included a total of 95 COVID-19 survivors with a median age of 62 (IQR 53–69) who had a complete functional status (PCFS grade 0) at baseline in this retrospective observational study. At 6-month follow-up, 67 (70.5%) patients had a complete functional outcome (grade 0), 9 (9.5%) had a negligible limited function (grade 1), 12 (12.6%) had a mild limited function (grade 2), 7 (7.4%) had moderate limited function (grade 3). Univariable logistic regression analysis showed a significant association between the onset symptoms of muscle or joint pain and an increased risk of incomplete function (unadjusted OR 4.06, 95% CI 1.33–12.37). This association remained after adjustment for age and admission delay (adjusted OR 3.39, 95% CI 1.06–10.81, p = 0.039).

**Conclusions:**

A small proportion of discharged COVID-19 patients may have an incomplete functional outcome at a 6-month follow-up; intervention strategies are required.

**Supplementary Information:**

The online version contains supplementary material available at 10.1186/s12879-021-06970-3.

## Background

Coronavirus disease 2019 (COVID-19) due to severe acute respiratory syndrome coronavirus 2 (SARS-CoV-2) infection was firstly reported in Wuhan, China in December 2019 [1]. As of 9 September 2021, the COVID-19 pandemic has spread worldwide, affecting more than 220 million people and killing over four millions lives [2]. Aggregating studies have shown that most SARS-CoV-2 infection was mild and moderate, which seems to have a positive recovery rate [3–5]. Previous studies with short-term follow-up data showed that a few discharged COVID-19 patients were re-positive for SARS-nCoV-2 detected by reverse transcription-polymerase chain reaction (RT-PCR) analysis [6, 7]. Moreover, in addition to physical damage, some COVID-19 patients may suffer from psychological impairment including sleep disorder, depression and anxiety after discharge [8, 9]. Previous studies also showed that discharged COVID-19 patients might have incompletely absorbed computed tomography (CT) findings, and some may develop residual pulmonary fibrosis [10, 11]. Moreover, a retrospective study showed that more than half of the COVID-19 patients in the early convalescence phase had impaired diffusing-capacity, lower respiratory muscle strength, and lung imaging abnormalities [12]. Patients with other coronavirus infection like severe acute respiratory syndrome (SARS) or Middle East respiratory syndrome (MERS) may have long-term persistent radiographic abnormalities in their lungs [13, 14]. It is reasonable to imagine that some COVID-19 patients may have adverse functional outcomes despite recovery. To our knowledge, the follow-up advice for those testing positive for COVID-19 is lacking, and the long-term functional status in COVID-19 survivors remains poorly understood. We aimed to describe a six-month follow-up of the functional status of COVID-19 patients after discharge in this retrospective cohort study.

## Methods

### Study design, participants and data collection

In this retrospective single-center observational study, we collected the demographic and clinical data of laboratory-confirmed COVID-19 patients who had been consecutively admitted to the Tumor Center of Union Hospital (Wuhan, China) between 15 February and 14 March 2020. The extraction and analysis of baseline data regarding demographic and clinical characteristics were documented in our previous published literature [15, 16]. Severe COVID-19 was defined as fever or suspected respiratory infection, plus one of: respiratory rate > 30 breaths/min; severe respiratory distress; or SPO_2_ ≤ 93% on room air based on the interim guidance of the World Health Organization [17]. The discharge criteria were as follows: 1. Normal body temperature for more than three days; 2. Significantly improved respiratory symptoms, 3. Substantial lung inflammation absorption on chest CT image, 4. Two consecutive negative results of nucleic acid tests for SARS-CoV-2 from the respiratory samples separated by at least one day [18]*.* We obtained and clarified data by direct communication with attending physicians and the healthcare providers when data were missing or uncertain from the medical records. We excluded patients if they did not undergo a post-COVID-19 functional status scale (PCFS) interview at six-month follow-up after discharge or had a PCFS > 0 at baseline (one month before the onset of COVID-19 symptoms).

### Follow-up

Patients were followed-up at a 6-month after discharge. The PCFS was designed as a measure to focus on relevant aspects of daily life during follow-up in COVID-19 patients [19, 20]. Briefly, we asked four questions to our participants or their caregivers: 1. Can you live alone without any assistance from another person? 2. Are there any duties and/or activities at home or at work which you are no longer able to perform yourself? 3. Do you suffer from symptoms, pain, depression or anxiety? 4. Do you need to avoid or reduce duties and/or activities or spread these over time? Based on the answers to these questions, the PFCS grades (0, 1, 2, 3, 4) were generated (Table 1). Grade 0 reflects the absence of any functional limitation, grade 1 and 2 mirror negligible to mild functional limitation, while grade 3 and 4 reflect moderate to severe limitation of functional status [20]. Two trained authors (S.F. and H.L.) who were blinded to the baseline routine clinical data performed the structured interview with participants by one telephone *(*with 'hand-free' function*)* interview in a quiet room at the same time at six-month after discharge, based on the PCFS manual (version May 2020) [20]. In case of disagreement, a consensus was reached after team discussion. We assessed inter-rater agreement on a random sample using Cohen’s Kappa coefficient.

**Table 1:** Post-COVID-19 Functional Status Scale

PCFS scale grade	Description
0. No functional limitations	No symptoms, pain, depression or anxiety
1. Negligible functional limitations	All usual duties/activities at home or at work can be carried out at the same level of intensity, despite some symptoms, pain, depression or anxiety
2. Slight functional limitations	Usual duties/activities at home or at work are carried out at a lower level of intensity or are occasionally avoided due to symptoms, pain, depression or anxiety
3. Moderate functional limitations	Usual duties/activities at home or at work have been structurally modified (reduced) due to symptoms, pain, depression or anxiety
4. Severe functional limitations	Assistance needed in activities of daily living due to symptoms, pain, depression or anxiety: nursing care and attention are required

*PCFS* Post-COVID-19 Functional Status Scale

### Outcomes

Our primary outcome was the functional status of the COVID-19 patients at a six-month follow-up by using a PCFS interview [19].

### Statistics

We summarized continuous data with mean value with standard deviations or median value with interquartile range (IQR), and categorized data as counts with percentages. We used the t-test or Mann–Whitney test to compare the differences in continuous variables, and the chi-square test or Fisher's exact test to compare the differences in categorical variables as appropriate. To permit a comparison, we dichotomized patients into complete (PCFS = 0) and incomplete (PCFS > 0) functional status at six-months follow-up after discharge. We included potentially significant variables if p ≤ 0.2 by univariable analysis into the multivariable logistic regression model, to investigate the factors for the incomplete functional (PCFS > 0). All statistics were performed using SPSS for windows 22.0 (IBM, Inc, USA).

## Results

**Fig. 1:**
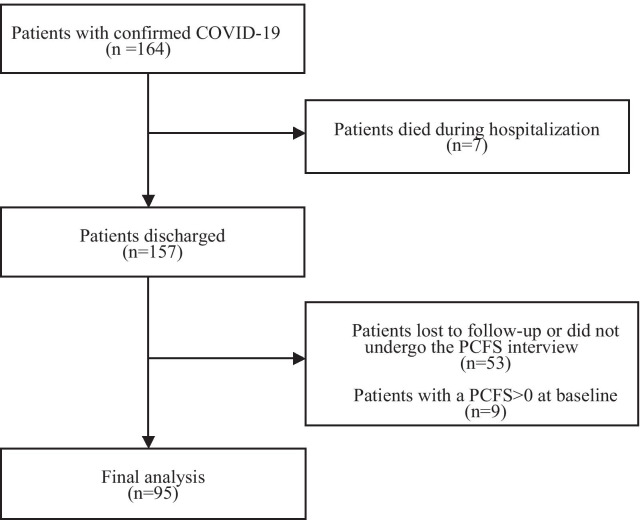
Flow chart of patients selection. *COVID-19* coronavirus disease 2019. *PCFS* post-COVID-19 functional status scale

We consecutively enrolled a total of 164 patients with laboratory-confirmed COVID-19 between 15 February and 14 March 2020. After excluding seven non-survivors and 53 lost to follow-up or did not undergo the PCFS interview, 104 patients (53 [50.9%] male) with a median age of 62 (IQR 54–70] participated in the follow-up. Patients with and without the PCFS interview were similar in age (63 [54–70] vs 62 [52–69], p = 0.493), to be male (53 [51.0%] vs 24 [45.3%], p = 0.501), and admission delay (13 [7–20] days vs 14 [9–21], p = 0.157). After further excluding three (2.9%) patients with grade 3 and six (5.8%) patients with grade 4 at baseline, we included 95 patients with a baseline PCFS = 0 in the final analysis (Fig. 1).

Table 2 shows the demographics and clinical characteristics of the study population. The inter-rater reliability for baseline PCFS interview was 0.68 (95% CI 0.46–0.90); for PCFS interview 6-month after discharge 0.79 (95% CI 0.65–0.93). All patients had no recurrence of symptoms or radiological findings, and there were no reported new stroke events or other major illness or death during 6-month follow-up. At 6-month follow-up, 67 (70.5%) patients had a complete functional outcome (grade 0), 9 (9.5%) had a negligible limited function (grade 1), 12 (12.6%) had a mild limited function (grade 2), and 7 (7.4%) had moderate limited function (grade 3). The differences in the demographics and clinical characteristics between patients with PCFS = 0 and PCFS > 0 at 6-month follow-up are shown in Table 3. Compared to those with PCFS = 0, patients with PCFS > 0 trended to be younger (60 [49–69] vs 64 [56–69], p = 0.164), more likely to had onset symptoms of muscle or joint pain (9 [32.1%] vs 7 [10.4%], p = 0.01), and had shorter onset-admission delay (9 days [6–18] vs 14 [10–20], p = 0.04).

**Table 2:** Baseline characteristics of study participants

	Total (n = 95)
Age, (y) median, (IQR)	62 (53–69)
Male, n (%)	50 (52.6)
Current smoker, n (%)	10 (10.5)
Regular drinker, n (%)	2 (2.1)
Hypertension, n (%)	27 (28.4)
Diabetes, n (%)	14 (14.7)
COPD, n (%)	6 (6.3)
Cardio-cerebrovascular disease, n (%)	16 (12.7)
Tumor, n (%)	7 (7.4)
Immunosuppressives, n (%)	2 (2.1)
Renal impairment, n (%)	11 (11.6)
Wet market exposure, n (%)	1 (1.1)
Clinical symptoms	
Fever, n (%)	69 (72.6)
Dry cough, n, (%)	62 (65.3)
Productive cough, n (%)	11 (11.6)
Fatigue, n (%)	35 (36.8)
Muscle or joint ache, n (%)	16 (16.8)
Thoracalgia, n (%)	16 (16.8)
Sore throat, n (%)	14 (14.7)
Diarrhea, n (%)	9 (9.5)
Catarrh, n (%)	5 (5.3)
Anorexia, n (%)	28 (29.5)
Short of breath, n (%)	33 (34.7)
Headache, n (%)	14 (14.7)
Routine blood examinations	
Decreased leucocytes, n (%)	5 (5.3)
Decreased lymphocytes, n (%)	27 (28.4)
Decreased hemoglobin, n (%)	24 (25.3)
Decreased platelets, n (%)	5 (5.3)
ALT or AST > 40U/L	37 (29.4)
Chest CT findings, n (%)	
Unilateral pneumonia, n (%)	16 (16.8)
Bilateral pneumonia, n (%)	55 (57.9)
Multiple mottling and ground-glass opacity, n (%)	24 (25.3)
Treated with steroid, n (%)	10 (10.5)
Antiviral, n (%)	93 (97.9)
Severe COVID-19, n (%)	13 (13.7)
Admission delay, (day) median, (IQR)	14 [8–21]

Decreased means below the lower limit of the normal range. Leucocytes (× 10^9^/L; normal range 3.5–9.5); Lymphocytes (× 10^9^/L; normal range 1.1–3.2); Platelets (× 10^9^/L; normal range 125.0–350.0); Hemoglobin (g/L; normal range 130.0–175.0)

*COVID-19* coronavirus disease 2019; *SD* standard deviation; *COPD* chronic obstructive pulmonary disease; *IQR* interquartile range; *ALT* alanine transaminase (U/L; normal range 0–40); *AST* alanine aminotransferase (U/L; normal range 0–40); *CT* computed tomography

**Table 3:** Baseline characteristics between COVID-19 survivors with complete and incomplete functional status at 6-months follow-up

	Complete function (n = 67)	Incomplete function (n = 28)	p-value
Age, (y) median, (IQR)	64 (56–69)	60 (49–69)	0.164
Male, n (%)	35 (52.2)	15 (53.6)	0.906
Current smoker, n (%)	6 (9.0)	4 (14.3)	0.685
Regular drinker, n (%)	1(1.5)	1(3.6)	> 0.999
Hypertension, n (%)	20 (29.9)	7 (25.0)	0.633
Diabetes, n (%)	10 (14.9)	4 (14.3)	> 0.999
COPD, n (%)	5 (7.5)	1 (3.6)	0.667
Cardio-cerebrovascular disease, n (%)	8 (11.9)	4 (14.3)	> 0.999
Tumor, n (%)	4 (6.0)	3 (10.7)	0.707
Immunosuppressives, n (%)	1 (1.5)	1 (3.6)	> 0.999
Renal impairment, n (%)	10 (14.9)	1 (3.6)	0.220
Wet market exposure, n (%)	1 (1.5)	0 (0)	> 0.999
Clinical symptoms			
Fever, n (%)	48 (71.6)	21 (75.0)	0.738
Dry cough, n, (%)	44 (65.7)	18 (64.3)	0.897
Productive cough, n (%)	7 (10.4)	4 (14.3)	0.856
Fatigue, n (%)	25 (37.3)	10 (35.7)	0.883
Muscle or joint ache, n (%)	7 (10.4)	9 (32.1)	0.010
Thoracalgia, n (%)	10 (14.9)	6 (21.4)	0.440
Sore throat, n (%)	11 (16.4)	3 (10.7)	0.691
Diarrhea, n (%)	8 (11.9)	1 (3.6)	0.376
Catarrh, n (%)	3 (4.5)	2 (7.1)	0.979
Anorexia, n (%)	18 (26.9)	10 (35.7)	0.388
Short of breath, n (%)	22 (32.8)	11 (39.3)	0.547
Headache, n (%)	11 (16.4)	3 (10.7)	0.691
Routine blood examinations			
Decreased leucocytes, n (%)	5 (7.5)	0 (0)	0.317
Decreased lymphocytes, n (%)	19 (28.4)	8 (28.6)	0.983
Decreased hemoglobin, n (%)	17 (25.4)	7 (25.0)	0.970
Decreased platelets, n (%)	3 (4.5)	2 (7.1)	0.979
ALT or AST > 40U/L	24 (35.8)	9 (32.1)	0.731
Chest CT findings, n (%)			
Unilateral pneumonia, n (%)	13 (19.4)	3 (10.7)	
Bilateral pneumonia, n (%)	36 (53.7)	19 (67.9)	
Multiple mottling and Ground-glass opacity, n (%)	18 (26.9)	6 (21.4)	0.407
Treated with steroid, n (%)	9 (13.4)	1 (3.6)	0.289
Antiviral, n (%)	66 (98.5)	27 (96.4)	> 0.999
Severe COVID-19, n (%)	10 (14.9)	3 (10.7)	0.828
Onset to admission, (day) median, (IQR)	14 [10–20]	9 [6–18]	0.04

Decreased means below the lower limit of the normal range. Leucocytes (× 10^9^/L; normal range 3.5–9.5); Lymphocytes (× 10^9^/L; normal range 1.1–3.2); Platelets (× 10^9^/L; normal range 125.0–350.0); Hemoglobin (g/L; normal range 130.0–175.0)

*COVID-19* coronavirus disease 2019; *SD* standard deviation; *COPD* chronic obstructive pulmonary disease; *IQR* interquartile range; *ALT* alanine transaminase (U/L; normal range 0–40); *AST* alanine aminotransferase (U/L; normal range 0–40); *CT* computed tomography

In univariable logistic regression analysis, onset symptoms of muscle or joint pain (unadjusted OR 4.06, 95% CI 1.33–12.37) were associated with an increased risk of having a PCFS > 0 at 6-month follow-up. We found a negative association between the onset-admission delay and a PCFS > 0 at six-month follow-up (unadjusted OR 0.95, 95% CI 0.89–1.00). After adjustment for age, onset symptoms of muscle or joint pain (adjusted OR 4.07 95% CI 1.32–12.54, p = 0.015) remained significantly associated with an increased risk of having a PCFS > 0 at 6-month follow-up. In the multivariable regression analysis, onset symptoms of muscle or joint pain remained significantly associated with an increased risk of incomplete functional status (adjusted OR 3.39 95% CI 1.06–10.81, p = 0.039). The association between the onset-admission delay and having a PCFS > 0 was lost in the multivariable regression model (Table 4).

**Table 4:** Risk factors for incomplete function status at 6-months follow-up

	Univariable		Age-adjusted		Multivariable	
	OR (95%CI)	p-value	OR (95%CI)	p-value	OR (95%CI)	p-value
Age	0.98 [0.95–1.01]	0.219	–	–	0.98[0.95–1.02]	0.259
Muscle or joint pain	4.06[1.33–12.37]	0.014	4.07[1.32–12.54]	0.015	3.39[1.06–10.81]	0.039
Admission delay	0.95 [0.89–1.00]	0.061	0.95[0.89–1.00]	0.065	0.96 [0.90–1.02]	0.163

Categorical variables are defined as 1 = yes, 0 = no

To be representative for patients with non-severe COVID 19, we performed a separate analysis by limited on those without severe COVID-19 (n = 82). Additional file 1: Table S1 summarizes the differences in baseline characteristics between patients with and without incomplete functional status at six-month follow-up. This separate analysis did not alter the association between joint or muscle pain and incomplete function. (unadjusted OR 4.0, 95% CI 1.21–13.18, p = 0.023, age-adjusted OR 4.14, 95% CI 1.23–13.90, p = 0.022, multivariate OR 3.46, 95% CI 0.99–12.07, p = 0.05).

## Discussion

The most important finding of the present study was that a small proportion of COVID-19 survivors may have an incomplete function status at a six-month follow-up after discharge. A previous study found that a considerable proportion of COVID-19 survivors without critical cases still had radiological and physiological abnormalities at three months after discharge [21]. Our study adds to findings of the previous study by incorporating insights into the functional outcome with a longer-term follow-up data. Several recently published literatures showed that a majority of COVID-19 survivors experienced COVID-19 related symptoms or functional limitations up to six months [22–24]. Different populations and assessing methods may account for the discrepancies among different studies. For example, one study also included patients with suspect COVID-19 assessed using an online panel [24], whereas trained physicians conducted a face-to-face interview in another study [22]. More studies with a longer follow-up are needed to better understand the important question for clinicians and the public: will patients recovered from COVID-19 have long-term sequelae?

In our cohort, COVID-19 survivors with the onset symptoms of joint or muscle pain were at an increased risk of having incomplete function status at six-month after discharge. In line with our finding, a previous study of 158 hospitalized COVID-19 patients showed that the symptoms of muscle or joint pain were significantly associated with the trend of intensification of COVID-19 (3/30% vs 3/128, p = 0.048) [25]. The associated muscle pain is one of the most frequent causes of pain in SARS-nCoV-2 infection. For example, a previous meta-analysis of ten observational studies showed that nearly 36% of COVID-19 patients had myalgia as one of the most common onset symptoms [26]. Although previous studies have suggested that the onset symptoms of muscle pain do not seem to increase with COVID-19 severity [3, 16, 27], in patients with abnormal chest radiographic findings, myalgia appeared to be an important risk factor for the severity of the overall disease [28]. The upregulation of the proinflammatory cytokines such as interleukin-6 during viral infection may cause muscle and joint pain [29]. Some researchers believe that myalgia in COVID-19 patients might mirror the systematic inflammation and cytokine response [30]. As SARS-CoV-2 infection induces robust immunologic complications like cytokine storm, elevated cytokine levels such as interleukin-6, interleukin-10, and tumor necrosis factor-α might occur, especially in patients with a moderate or severe disease course [31, 32]. This hypothesis was supported by a previous observational study that showed COVID-19 patients with muscle injury had manifestations of increased inflammatory response and blood coagulation function [33]. Although our study cannot provide comparative data to determine the effects of COVID-19 on the long-term functional outcome, our findings will contribute to determining COVID-19 at initial stages and suggesting medical intervention in a timely manner.

Our data suggest that the inter-rater reliability of the PCFS interview was satisfactory. Moreover, both raters reported no significant difficulties with scale interpretation, indicating that the PCFS is a simple and feasible approach to monitor the course of symptoms and the impact of symptoms on the functional status of COVID-19 survivors. Previous studies have shown that the functional impairment checklist is reliable, valid and responsive to changes in symptom and disability as a consequence of SARS, suggesting it may provide a means of assessing health-related quality of life outcomes in a longitudinal follow-up [34].

### Limitation

First, this is a small sample-sized retrospective observational study without a predefined protocol. Due to the likely self-selection bias by covering only those undergo the post-COVID-19 survey, our findings need to be interpreted with caution and validated in further large-sample studies. Second, since most of our cohort did not experience severe COVID-19, our findings may not be generalized for patients with severe COVID-19. Third, although we only included patients with complete baseline functional status in our final analysis, we cannot ensure that the functional decline was due to COVID-19. However, when responded to Q3 (Do you suffer from symptoms, pain, depression or anxiety?) our patients reported these symptoms are caused by or mostly related to COVID-19. Fourth, we did not validate the PCFS assessment with other well-validated tools such as six-minutes walking exercise and Saint-Jeorge respiratory scale. Several recent studies have shown that the PCFS is a validated scale for evaluating 3 to 6-month functional outcomes in COVID-19 patients [24, 35]. Future studies are needed to benchmark PCFS with other validated tools in Chinese COVID-19 patients. Results from the LEOSS registry (Lean European Open Survey on SARS-CoV-2 Infected Patients; https://LEOSS.net) will better address the long-term functional outcomes.

## Conclusions

The present study indicated that a small proportion of COVID-19 survivors may have incomplete function status at six-month follow-up, and the risk of incomplete function is higher among patients presenting at baseline with muscle or joint pain. Such patients may benefit from follow-up rehabilitation programs.

## Supplementary Information

**Additional file 1: Table 1.** Baseline characteristics between COVID-19 non-severe survivors with complete and incomplete functional status at 6-months follow-up.

## Data Availability

The datasets generated during and/or analyzed during the current study are available from the corresponding author at xieheliunan1984@fjmu.edu.cn on reasonable request.

## Abbreviations

COVID-19: Coronavirus disease 2019
PCFS: Post-COVID-19 Functional Status Scale
SARS-CoV-2: Severe acute respiratory syndrome coronavirus 2
RT-PCR: Reverse transcription-polymerase chain reaction
MERS: Middle East respiratory syndrome
