# Supplementary material for: Six-month follow-up of functional status in discharged patients with coronavirus disease 2019
Source: BMC Infect Dis. 2021 Dec 20;21:1271. doi: 10.1186/s12879-021-06970-3 (PMC8686090; doi:10.1186/s12879-021-06970-3)
Supplement: Supplementary file 1 — Additional file 1: Table 1. Baseline characteristics between COVID-19 non-severe survivors with complete and incomplete functional status at 6-months follow-up. [file 12879_2021_6970_MOESM1_ESM.docx]

**Supplemental Table 1: Baseline characteristics between COVID-19 non-severe survivors with complete and incomplete functional status at 6-months follow-up**

|  | Complete funtion  (n = 57) | Incomplete function  (n = 25) | P-value |
| --- | --- | --- | --- |
| Age, (y) median, (IQR) | 64 (54-69) | 57 (48-68) | 0.137 |
| Male, n (%) | 29 (50.9) | 12 (48.0) | 0.810 |
| Current smoker, n (%) | 6 (10.5) | 4 (16.0) | 0.741 |
| Regular drinker, n (%) | 1(1.8) | 1(4.0) | >0.999 |
| Hypertension, n (%) | 16 (28.1) | 7 (28.0) | 0.995 |
| Diabetes, n (%) | 7 (12.3) | 4 (16.30) | 0.918 |
| COPD, n (%) | 2 (3.5) | 1 (4.0) | >0.999 |
| Cardio-cerebrovascular disease, n (%) | 5 (8.8) | 4 (16.0) | 0.562 |
| Tumor, n (%) | 3 (5.3) | 2 (8.0) | >0.999 |
| Immunosuppresives, n (%) | 0 (0) | 1 (4.0) | 0.305 |
| Renal impairment, n (%) | 7 (12.3) | 1 (4.0) | 0.448 |
| Wet market exposure, n (%) | 0 (0) | 0 (0) | / |
| Clinical symptoms |  |  |  |
| Fever, n (%) | 39 (68.4) | 18 (72.0) | 0.746 |
| Dry cough, n, (%) | 36 (63.2) | 17 (68.0) | 0.673 |
| Productive cough, n (%) | 7 (12.3) | 3 (12.0) | 0.971 |
| Fatigue, n (%) | 23 (40.4) | 9 (36.0) | 0.710 |
| Muscle or joint ache, n (%) | 6 (10.5) | 8 (32.0) | 0.017 |
| Thoracalgia, n (%) | 9 (15.8) | 5 (20.0) | 0.641 |
| Sore throat, n (%) | 8 (14.0) | 3 (12.0) | >0.999 |
| Diarrhea, n (%) | 6 (10.5) | 1 (4.0) | 0.586 |
| Catarrh, n (%) | 3 (5.3） | 2 (8.0) | >0.999 |
| Anorexia, n (%) | 16 (28.1) | 9 (36.0) | 0.476 |
| Short of breath, n (%) | 17 (29.8) | 10 (40.0) | 0.367 |
| Headache, n (%) | 9 (15.8) | 3 (12.0) | 0.914 |
| Routine blood examinations  Decreased leucocytes, n (%)  Decreased lymphocytes, n (%)  Decreased hemoglobin, n (%)  Decreased platelets, n (%) | 3 (5.3)  14 (24.6)  12 (21.1)  1 (1.8) | 0 (0)  6 (24.0)  5 (20.0)  2 (8.0) | 0.549  0.957  0.914  0.454 |
| ALT or AST > 40U/L | 19 (33.3) | 7 (28.0) | 0.633 |
| Chest CT findings, n (%)  Unilateral pneumonia, n (%)  Bilateral pneumonia, n (%)  Multiple mottling and Ground-glass opacity, n (%) | 12 (21.1)  32 (56.1)  13 (22.8) | 3 (12.0)  17 (68.0)  5 (20.0) | 0.535 |
| Treated with steroid, n (%) | 6 (10.5) | 0 (0) | 0.170 |
| Antiviral, n (%) | 56 (98.2) | 24 (96.0) | >0.999 |
| Onset to admission, (day) median, (IQR) | 14 [10-20] | 10 [6-19] | 0.061 |

Abbreviations: COVID-19 = coronavirus disease 2019; SD = Standard deviation; COPD = Chronic obstructive pulmonary disease; IQR = Interquartile range; ALT = Alanine transaminase (U/L; normal range 0 - 40); AST = Alanine aminotransferase (U/L; normal range 0 - 40); CT = Computed tomography; Decreased means below the lower limit of the normal range. Leucocytes (× 10^9^/L; normal range 3.5-9.5); Lymphocytes (× 10^9^/L; normal range 1.1-3.2); Platelets (× 10^9^/L; normal range 125.0–350.0); Hemoglobin (g/L; normal range 130.0-175.0)
